# A Simplified Physical Model for the Sensitivity–Pressure Relationship in Textile-Based Piezoresistive Sensors

**DOI:** 10.3390/s26103081

**Published:** 2026-05-13

**Authors:** Kai Shi, Yanan Tao, Xuechun Xu, Zhehao Xiong, Jianjun Shi, Ying Guo

**Affiliations:** 1 College of Physics, Donghua University, Shanghai 201620, China; 2242566@mail.dhu.edu.cn (K.S.); 1239157@mail.dhu.edu.cn (Y.T.); 240130125@mail.dhu.edu.cn (Z.X.); 2 College of Textiles, Donghua University, Shanghai 201620, China; 2240289@mail.dhu.edu.cn; 3 State Key Laboratory of Surface Physics, Fudan University, Shanghai 200438, China; 4 International Institute for Intelligent Nanorobots and Nanosystems, College of Intelligent Robotics and Advanced Manufacturing, Fudan University, Shanghai 200438, China; 5 Zhejiang Key Laboratoy of Extreme Environment Functional Materials, Yiwu Research Institute of Fudan University, Yiwu 322099, China

**Keywords:** flexible pressure sensor, textile-based sensor, piezoresistive effect, physical model, structure–property relationship

## Abstract

Textile-based flexible pressure sensors have attracted considerable attention in wearable sensing applications due to their good comfort and mechanical compatibility. However, their sensitivity usually exhibits a nonlinear dependence on pressure, while a compact analytical framework with interpretable physical parameters is still lacking. In this work, a simplified physical model based on lumped effective parameters was established based on the evolution of fiber–conductive particle contacts, and an expression describing the sensitivity–pressure relationship was derived. The model indicates that the sensitivity is mainly governed by an electrical parameter *α* and a mechanical parameter ratio $E_{b}/E_{x}$, and captures the dominant nonlinear decrease in sensitivity with increasing pressure. To verify the applicability of the model, the effects of conductive particle loading, filler type, surface treatment, sensing-layer area, weave structure, and layer number on the sensor response were systematically investigated. In addition, comparison between model-based calculation and experiment in the low- and medium-pressure range gave RMSE values of 0.0040 and 0.0056, and MRE values of 27.6% and 13.4% for the single-layer and four-layer structures, respectively. These results show that the proposed framework captures the main trends of the sensitivity–pressure behavior and provides a physically interpretable basis for discussing how structural and material factors regulate sensor response. This work offers a useful framework for understanding the structure–property relationship of textile-based piezoresistive pressure sensors and may provide preliminary guidance for the design of customized sensors in wearable healthcare and soft robotics applications.

## 1. Introduction

With the development of flexible electronics and wearable technologies, the demand for pressure sensors that combine good mechanical compliance with stable electrical response has continued to increase [1,2,3,4,5,6,7,8]. Textile-based flexible pressure sensors, constructed on fibrous textile substrates, offer advantages such as softness, breathability, light weight, and ease of large-area integration. They are therefore regarded as promising devices for long-term and comfortable human signal monitoring, with broad application potential in health monitoring, human–machine interaction, and soft robotics [9,10,11,12,13,14]. In these applications, sensors are required not only to exhibit high sensitivity but also to maintain predictable and stable responses within a certain pressure range. For this reason, the sensitivity–pressure relationship has become a key issue in device design and performance evaluation [15].

Recent studies on textile-based flexible pressure sensors have mainly focused on material systems and structural design. Functional materials such as carbon nanotubes, graphene, conductive polymers, and their composites have been introduced to construct conductive networks and improve sensing performance [16,17,18,19,20,21]. Meanwhile, the pressure response has been regulated by changing textile structures, such as weave type, number of layers, pore structure, or multiscale composite architectures [22,23,24,25,26]. These sensors commonly exhibit pronounced nonlinear piezoresistive behavior, with relatively high sensitivity in the low-pressure region and gradually reduced sensitivity as pressure increases. However, most existing studies still focus on device performance comparison, where the response is commonly described by sensitivity values, response curves, or empirical fitting forms such as power-law or exponential relations. The physical factors governing sensitivity variation are not always explicitly clarified [27,28,29,30,31].

Another practical difficulty is the limited comparability of reported sensitivity values. Depending on whether resistance, current, capacitance, or voltage is used for calculation, the same underlying pressure response may yield different numerical sensitivity values. Therefore, sensitivity alone may not always provide a sufficiently intuitive or physically meaningful basis for evaluating sensor performance. This issue suggests the need to move beyond isolated sensitivity values and identify more fundamental factors that determine the pressure response.

From a mechanistic point of view, the electrical response of textile-based piezoresistive pressure sensors is generally associated with the evolution of conductive networks under external loading, including fiber deformation, changes in contact state, and the formation or enhancement of conductive pathways [32,33,34]. Applied pressure can increase the effective contact area between conductive layers, thereby reducing the overall resistance [35]. In fibrous or nanostructured systems, the rapid increase of microscopic contact points usually contributes to high sensitivity in the low-pressure region, whereas the gradual saturation of contact growth leads to sensitivity decay at higher pressures [36]. In addition, different conductive materials may alter the piezoresistive response by changing the contact behavior between conductive particles [37,38,39].

Although previous studies have provided useful mechanistic insights, most analyses remain qualitative, semi-quantitative, or device-specific. Compact analytical frameworks with interpretable parameters are still limited, especially for textile-based piezoresistive systems involving coupled structural and electrical effects. As a result, the influence of processing and structural variables, such as conductive particle loading, textile pretreatment, and weave selection, is often judged mainly through empirical comparison rather than within a unified parameter framework.

Based on these considerations, a simplified lumped-parameter physical model is proposed in this work. Rather than serving as a fully predictive constitutive model, it is intended as an explanatory and semi-quantitative framework for interpreting the dominant sensitivity–pressure trends in textile-based piezoresistive sensors. Starting from fiber compression and conductive-layer contact evolution, the model connects macroscopic resistance variation with microscopic contact-area change through simplified geometric and mechanical assumptions. It suggests that the sensitivity is mainly governed by an effective electrical parameter related to contact-resistance variation and an effective modulus ratio characterizing the compressive response of the textile structure.

To verify the applicability of the framework, a series of textile-based pressure sensors with different structural configurations were fabricated and tested. By varying conductive loading, filler type, substrate treatment, sensing-layer geometry, weave type, and layer number, the corresponding effects on the sensitivity–pressure relationship were evaluated. The main contributions of this work are as follows: (1) a simplified explanatory model for the sensitivity–pressure relationship is proposed; (2) the effects of multiple structural parameters on sensor response are systematically investigated; and (3) the main response trends are semi-quantitatively interpreted through effective model parameters. The sources of materials, fabrication procedures, and testing methods are described in Section 2.

## 2. Materials and Methods

**Figure 1:**
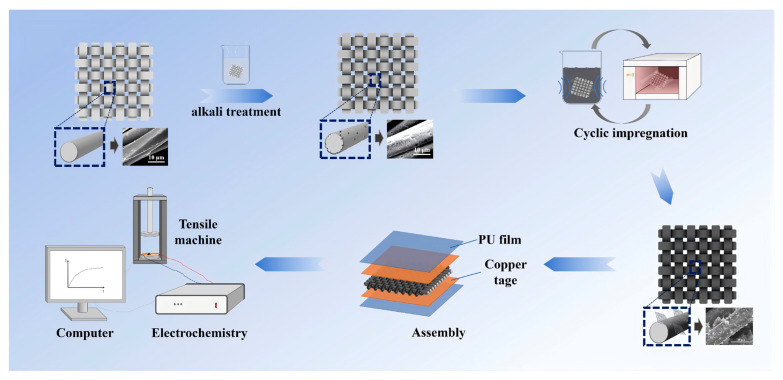
Schematic illustration of the fabrication process of the textile-based piezoresistive pressure sensor, including alkali treatment, ultrasonic dip-coating, drying, sensor assembly, and electrical measurement.

The overall fabrication process of the textile-based piezoresistive pressure sensor is schematically illustrated in Figure 1.

### 2.1. Materials

Polydimethylsiloxane precursor (PDMS, MW 770) was purchased from Alfa Aesar (Shanghai, China). Poly(ethylene terephthalate) (PET) fabrics, including satin, plain, and twill weaves with an areal density of 120 g/m^2^, were supplied by Zhejiang Miandu Textile Co., Ltd. (Huzhou, China). The 209 detergent solution (analytical grade) was obtained from Guangzhou Wangnilai Co., Ltd. (Guangzhou, China). Ethanol (AR, ≥99.7%) was purchased from Changzhou Hongsheng Fine Chemical Co., Ltd. (Changzhou, China). Reduced graphene oxide (r-GO, average particle size of about 3 μm and thickness < 100 nm) was obtained from Guangdong Jiazhaoye Materials Co., Ltd. (Guangzhou, China). Hydroxylated multi-walled carbon nanotubes (CNTs, diameter 3–20 nm, length < 30 μm) were purchased from Chengdu Organic Chemicals Co., Ltd. (Chengdu, China). Sodium hydroxide (NaOH, analytical grade) was obtained from Sinopharm Chemical Reagent Co., Ltd. (Shanghai, China). Deionized water was used throughout the experiments.

### 2.2. Preparation of Conductive Fabrics

The PET fabrics were first ultrasonically cleaned in a 2 g/L 209 detergent solution for 30 min with a bath ratio of 1:50, then further cleaned in deionized water for 30 min with the same bath ratio, and finally dried in an oven at 80 °C. For alkali treatment, the pre-cleaned fabrics were immersed in a NaOH solution at 90 °C for 10 min, washed with deionized water until the pH approached neutral, and then dried at 80 °C.

The graphene suspension was prepared by dispersing graphene powder (3 g/L) in absolute ethanol, followed by ultrasonic treatment at room temperature for 2 h. PDMS precursor (30 g/L) was then added, and the mixture was further ultrasonicated for 1 h to obtain a uniformly dispersed graphene conductive suspension.

The CNT suspension was prepared by adding CNTs to absolute ethanol at a concentration of 2 wt%, followed by ultrasonic dispersion for 1 h and centrifugation at 2000 r/min for 20 min to obtain a stable dispersion. The NaOH solution used for alkali treatment had a concentration of 25 wt%.

The pretreated PET fabrics, either untreated or alkali-treated, were immersed in the conductive suspensions and subjected to ultrasonic dip-coating. Every 30 min, the fabrics were removed and dried at 60 °C for 15 min, which was defined as one dip-coating cycle. Unless otherwise specified, the fabrics were subjected to five dip-coating cycles to obtain stable conductive networks.

### 2.3. Sensor Fabrication

The conductive fabrics were cut into square samples with dimensions of 1 cm × 1 cm. Copper foil conductive tape was attached to both ends of each sample as electrodes to fabricate the pressure sensors. When the number of sensing-layers was taken as a variable, the conductive fabric pieces were stacked according to the designed layer number before electrode packaging.

### 2.4. Characterization and Measurement

The surface morphology of the fabrics was characterized using a field-emission scanning electron microscope (FE-SEM, S-4800, Hitachi, Tokyo, Japan). The electrical performance of the sensors was measured using a CH1660E electrochemical workstation. The measurement system consisted of the sensor sample, a laboratory-made loading device, and an AC power supply (CTP-2000K, Sunmen, Nanjing, China). The resistance variation of the sensor was recorded under different applied pressures. All reported sensitivity data were obtained from six independently fabricated samples under identical conditions, and the corresponding standard deviations are shown as error bars to reflect batch-to-batch variation and measurement repeatability.

## 3. Results and Discussion

To explain the typical nonlinear behavior of the sensitivity–pressure (*S*–*P*) relationship in textile-based pressure sensors, a simplified physical model is first established in this section to analyze the basic dependence of sensitivity on pressure. On this basis, a series of controlled experiments involving structural and material parameters was carried out to examine and discuss the main physical mechanisms revealed by the model. The experimental design is based on varying one key parameter at a time, while keeping the other conditions as consistent as possible, and comparing the resulting *S*–*P* curves under different conditions. The aim is to determine whether these variations can be reasonably interpreted in terms of the electrical parameter α and the mechanical parameter ratio $E_{b}/E_{x}$ introduced in the model, and thereby to evaluate the applicability of the model in understanding the pressure-sensing behavior of textile-based sensors. All sensor samples were fabricated using the dip-coating process described in Section 2. To facilitate understanding of the model derivation, the main variables used in this work are summarized in Nomenclature part.

### 3.1. Physical Model for the Sensitivity–Pressure Relationship

To explain the commonly observed sensitivity–pressure response in textile-based pressure sensors, a simplified physical model is established in this subsection. The basic idea of the model is to relate the measurable macroscopic electrical response, namely the resistance variation, to the microscopic structural deformation and contact evolution induced by external pressure. By introducing necessary and controlled idealized assumptions, the complex and random textile structure is reduced to a physical framework suitable for mechanical and electrical analysis, so that an analytical expression for sensitivity with clear physical meaning can be derived. It should be noted that the model is not intended to provide precise prediction for a specific device, but rather to serve as a theoretical framework for understanding the main sensing mechanism, interpreting the general trends observed in experiments, and guiding material and structural design.

#### 3.1.1. Basic Assumptions and Model Establishment

To capture the dominant physical processes in textile-based pressure sensing and to construct a model that can be treated analytically, the following assumptions are made.

**Figure 2:**
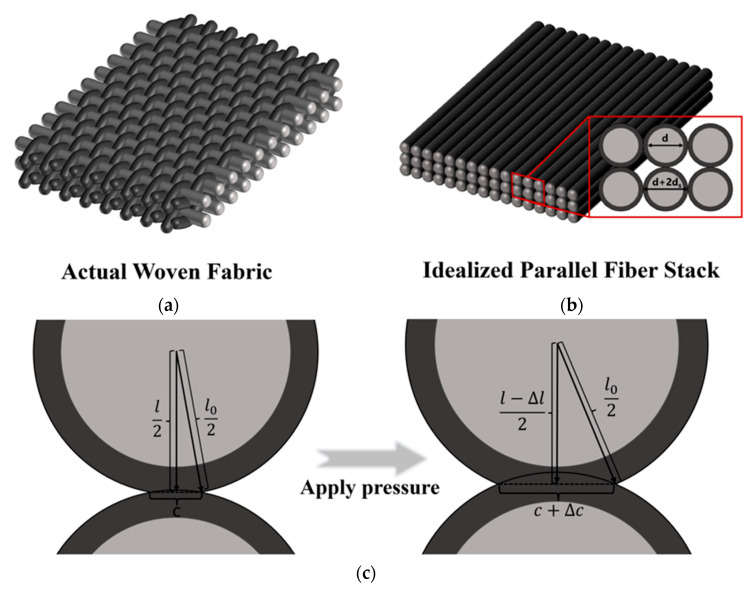

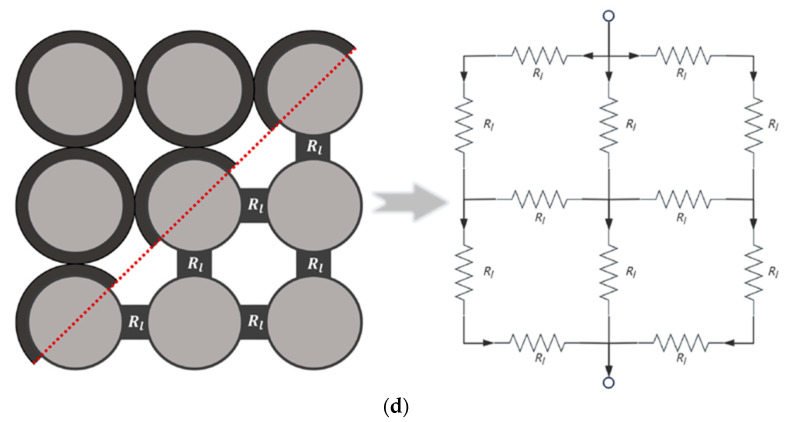
Schematic illustration of the structural model and sensing mechanism of the woven fiber sensor. (**a**) Actual woven fabric structure. (**b**) Idealized parallel fiber stack used for theoretical modeling. (**c**) Deformation of the fiber contact region under applied pressure. (**d**) Equivalent resistor network representing the conductive pathways in the fiber assembly.

First, the fibers constituting the textile sensing layer are treated as flexible cylindrical elements with the same diameter *d* and effective length *a*. They are assumed to be stacked approximately in parallel, forming an idealized structural unit with side length *a* and initial thickness *h*_0_ (Figure 2b).

Second, the conductive particles introduced by dip-coating, such as rGO or CNTs, are assumed to form a conductive layer with approximately uniform thickness *d_s_* on the fiber surface. This conductive coating is regarded as the main pathway for charge transport.

Third, the core sensing mechanism is assumed to be governed by the pressure-induced radial deformation of fibers. Under external pressure *P*, neighboring conductive coatings gradually come into contact and the contact area increases (Figure 2c). It is assumed that the evolution of this contact area is the dominant factor controlling the change in the overall resistance of the conductive network, while the intrinsic resistance variation of the fiber substrate itself is neglected.

Based on these assumptions, the pressure-sensing process can be divided into two coupled parts:

(i) A mechanical process: pressure → fiber deformation → contact area variation;
(ii) An electrical process: contact area variation → contact resistance variation → total resistance variation.

The following derivation is carried out based on the quantitative relations between these two processes.

#### 3.1.2. Relationship Between Structural Deformation and Contact Conductance

The mechanical process is considered first. For two adjacent parallel coated fibers, the initial center-to-center distance is denoted as *l*_0_. Under external pressure, this distance decreases to *l*. In the idealized cross-section, the effective contact width between the two coated fibers is denoted as *c*, and it is approximated as:

$$\;c\;=\;\sqrt{l_{0}^{2}{\;-\;l}^{2}}$$

where *l*_0_ = *d* + 2*d_s_*. Since *a* represents the effective fiber length within the representative unit, the corresponding inter-fiber contact area is expressed as:

$$\;A\;=\;a\;*\;c\;=\;a\sqrt{l_{0}^{2}{\;-\;l}^{2}}$$

This relation indicates that the contact area *A* is a function of the inter-fiber distance *l*. When the applied pressure changes by a small increment *dP*, the fiber undergoes a corresponding small radial deformation *dl*. Differentiation of the above geometric relation gives the relationship between the relative variation in contact area and the relative variation in the inter-fiber distance:

$$\;\frac{\mathrm{dA}}{A}=-\frac{\mathrm{ldl}}{l_{0}^{2}{\;-\;l}^{2}}$$

To relate the deformation *dl* to the macroscopic pressure increment *dP*, two effective elastic parameters are introduced. The first is the effective radial elastic modulus:

$$\;E_{x}\;=\;l\cdot \frac{\mathrm{dP}}{\mathrm{dl}},$$

which characterizes the overall stiffness of a single fiber together with its conductive coating under radial compression. The second is the effective through-thickness elastic modulus:

$$\;E_{b}\;=\;h\cdot \frac{\mathrm{dP}}{\mathrm{dh}},$$

which describes the macroscopic compressive response of the whole textile structure along the thickness direction.

It should be emphasized that *E_x_* and *E_b_* are not intrinsic material moduli in the conventional sense, but effective lumped parameters representing the radial compression stiffness of the fiber unit and the overall through-thickness compression stiffness of the textile structure. The value of *E_x_* mainly depends on the fiber substrate, coating thickness, and the radial stiffness of the composite structure, whereas *E_b_* reflects the macroscopic compression response of the textile architecture, including weave type, number of layers, and packing density. Experimentally, *E_b_* may be estimated from through-thickness compression tests of the textile, while *E_x_* may be obtained from radial compression or local mechanical characterization of coated fibers. In the present work, these quantities are introduced as effective mechanical descriptors rather than independently measured material constants. According to reported studies on flexible porous polymers and fibrous networks [40], the relevant moduli are typically within the kPa to 10^2^ kPa range, and thus an effective ratio $E_{b}/E_{x}$ on the order of 10^−2^ to 10^0^ is reasonable. Parameter analysis further suggests that a larger $E_{b}/E_{x}$ leads to a faster decay of sensitivity with increasing pressure, thereby affecting the overall nonlinearity of the *S*–*P* curve. By substituting these definitions into the differentiated geometric relation and rearranging the result (a detailed derivation is provided in the Supplementary Materials), the relative change in contact area can be expressed in a form that depends only on pressure variation and the effective mechanical parameters:

(1) $$\;\frac{\mathrm{dA}}{A}\;=\;\frac{1}{2\frac{E_{b}\cdot \mathrm{dP}}{{E_{x}}^{2}}+4\cdot \frac{E_{b}}{E_{x}}}$$

This expression serves as the key intermediate variable linking external pressure variation to the electrical response.

#### 3.1.3. Equivalent Circuit Model and Sensitivity Expression

From the electrical point of view, an increase in the inter-fiber contact area *A* leads to an increase in contact conductance and a decrease in contact resistance. To describe the overall electrical response of the textile, the complex conductive network is simplified into an equivalent circuit model (Figure 2d). The contact region between adjacent fibers is represented by an equivalent resistance *R_l_*, while the relatively small resistance of the conductive path along the fiber body is neglected in the first-order approximation. Under this model, the total resistance *R* of the textile can be expressed accordingly:

(2) $$\;{R\;=\;K}_{1}\cdot R_{l}{+K}_{2}$$

Here, K_1_ is a dimensionless geometric factor associated with the topology of contact points inside the textile, and K_2_ is a constant resistance term including contributions such as electrode contact. For a textile sensor with fixed structure and fabrication conditions, K_1_ and K_2_ can be regarded as constant during the measurement. The equivalent resistance R_l_ of a single contact region is further described by a lumped expression:

(3) $$\;R_{l}\;=\;\rho \frac{L}{A}+N\cdot R_{j}$$

where *ρ* is the effective resistivity of the conductive coating, *L* is the effective transport length, *A* is the inter-fiber contact area, *R_j_* is the representative inter-particle contact resistance, and *N* is an effective particle-contact factor. Equation (3) is a lumped description of one equivalent resistance element, in which the first term represents the bulk transport contribution and the second term reflects the particle-contact contribution.

According to the definition of sensitivity and by combining Equations (1)–(3), and assuming that the resistance variation under pressure is mainly caused by the evolution of contact area *A*, while *N*, *R_j_*, and *ρ* remain nearly unchanged under small deformation, the sensitivity expression can be obtained:

(4) $$\;S\;=\;-\frac{1}{\left[1+\frac{A}{\rho L}\left({\mathrm{NR}}_{j}{+K}_{2}/K_{1}\right)\right]}\cdot \left(\frac{1}{2\frac{E_{b}\mathrm{dP}}{E_{x}^{2}}+4\cdot \frac{E_{b}}{E_{x}}+1}\right)\cdot \frac{1}{\mathrm{dP}}$$

For convenience, a dimensionless electrical factor *α* is introduced to simplify the expression:

$$\;\alpha \;=\;\frac{1}{\left[1+\frac{A}{\rho L}\left({\mathrm{NR}}_{j}{+K}_{2}/K_{1}\right)\right]},$$

this factor ranges from 0 to 1 and reflects the weight of contact-resistance variation in the total resistance change. When the contact resistance dominates, *α* → 1, the sensitivity is mainly governed by contact evolution. When the constant resistance term cannot be neglected, *α* < 1, and the sensitivity is correspondingly weakened. By taking the initial pressure as zero and assuming that *α* remains approximately constant in the small-pressure region, a general analytical expression for the sensitivity–pressure relationship can be obtained:

(5) $$\;S\;=\;\frac{-\alpha }{2\frac{E_{b}}{E_{x}^{2}}P^{2}+\left(4\cdot \frac{E_{b}}{E_{x}}+1\right)P}$$

When the pressure variation range is limited or $E_{b}/E_{x}$ is relatively small, this expression can be further simplified into an inverse form:

(6) $$\;S\approx -\frac{\alpha }{\left(4\frac{E_{b}}{E_{x}}+1\right)P}\;=\;\frac{k}{P}$$

#### 3.1.4. Physical Meaning of Model Parameters

The final sensitivity expression decomposes the macroscopic electrical response into three parts with clear physical meaning: the electrical factor α, the mechanical parameter ratio $E_{b}/E_{x}$, and the nonlinear pressure-dependent term. This decomposition is useful for understanding how different materials, structures, and fabrication parameters affect sensing performance.

It should also be noted that *E_x_*, *E_b_*, and *α* are effective lumped parameters. In principle, *E_x_* may be obtained by applying radial compression to a coated fiber and measuring the corresponding deformation, while *E_b_* may be estimated from through-thickness compression tests of the textile structure. However, rigorous independent measurement of these parameters was not performed in the present work due to experimental limitations. Therefore, in this study, they should be regarded as effective descriptors for semi-quantitative comparison and mechanistic interpretation, rather than as directly measured input parameters for strict prediction.

Based on the above theoretical analysis, the basic relationship between sensitivity and pressure can be obtained. To verify the applicability of this relationship to actual textile sensors, the fundamental functional relation used in the model is first examined experimentally.

### 3.2. Preliminary Validation of the Model

**Figure 3:**
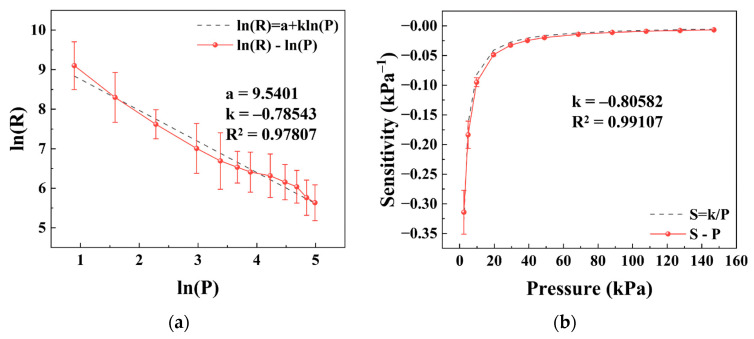
Experimental verification of the fundamental relationship used in the model derivation. (**a**) Log–log plot of resistance as a function of applied pressure with linear fitting according to ln(*R*) = a + *k*ln(*P*). (**b**) Sensitivity as a function of pressure, where the dashed line represents the theoretical relationship *S* = *k*/*P*. Data are presented as the mean ± standard deviation from six independently fabricated samples under identical conditions (*n* = 6).

According to the analysis in Section 3.1, the sensitivity is approximately proportional to the inverse of pressure, i.e., *S* ∝ 1/*P*, and the resistance–pressure relationship is expected to follow a power-law form. To verify this prediction, the experimental data were first transformed into logarithmic coordinates, as shown in Figure 3a. In the ln(*R*)–ln(*P*) plot, the data exhibited a clear linear relationship over a relatively wide pressure range. Linear fitting yielded a slope of *k* = −0.79 with a coefficient of determination *R*^2^ > 0.97, indicating that the resistance variation with pressure can be reasonably described by a power-law function:

$$\;R\;\propto {\;P}^{k}.$$

Based on this relation, the sensitivity can be further expressed as *S* = *k*/*P*. The experimental data were then fitted using this expression, and the results are shown in Figure 3b. The fitted slope was *k* = −0.81 with *R*^2^ > 0.99, which is consistent with the result obtained from the ln(*R*)–ln(*P*) fitting.

These results confirm that the proposed analytical relation captures the main feature of the sensor response. The sensitivity–pressure behavior can therefore be approximated by an inverse relation within the considered pressure range, providing a basis for the subsequent analysis.

### 3.3. Effect of Conductive Particle Loading

**Figure 4:**
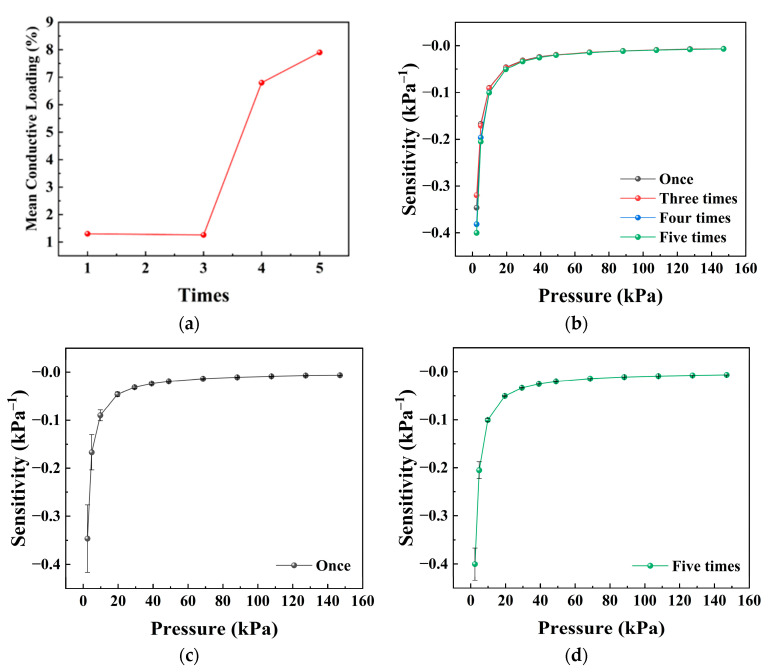
Effect of conductive particle loading regulated by dip-coating cycles on the pressure sensitivity of the textile-based sensor. (**a**) Mean conductive loading as a function of dip-coating cycles. (**b**) Sensitivity–pressure curves of sensors prepared with different dip-coating cycles. (**c**) Sensitivity–pressure curve with error bars for the sensor prepared by one dip-coating cycle. (**d**) Sensitivity–pressure curve with error bars for the sensor prepared by five dip-coating cycles. Data are presented as the mean ± standard deviation from six independently fabricated samples under identical conditions (*n* = 6).

The effect of conductive particle loading on the sensitivity–pressure behavior was investigated by varying the number of dip-coating cycles, as shown in Figure 4. As the number of coating cycles increases, the overall conductivity of the textile is improved, and the sensitivity in the low-pressure region is correspondingly enhanced.

From the model perspective, increasing the loading of conductive particles improves the coverage and continuity of the conductive layer on the fiber surface, and strengthens the electrical connections between neighboring conductive particles. This effect is mainly reflected in an increase in the electrical factor α, with the most direct contribution arising from the reduction in the effective resistivity ρ of the conductive coating. In addition, higher loading may also change the static particle-contact contribution within the conductive coating, which is reflected in the effective parameter *N*.

According to the sensitivity expression, an increase in α leads to higher sensitivity, especially in the low-pressure region. The experimental results are consistent with this analysis, indicating that adjusting the dip-coating cycles provides an effective way to tune the electrical factor α and thereby regulate the sensor response. This interpretation also suggests that the influence of conductive-particle loading should be understood as a coupled effect of coating resistivity, particle-contact contribution, and contact-area evolution, rather than as a simple monotonic effect of increasing particle number.

### 3.4. Effect of Conductive Filler Type

**Figure 5:**
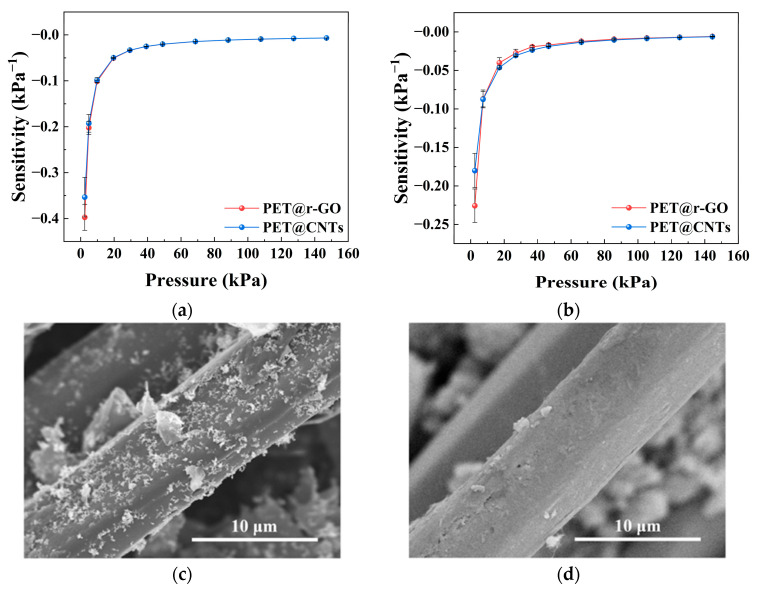
Effect of conductive filler type on the pressure sensitivity of the textile-based sensor. (**a**) Sensitivity–pressure curves of PET@r-GO and PET@CNTs under an initial preload of 0 g. (**b**) Sensitivity–pressure curves of PET@r-GO and PET@CNTs under an initial preload of 25 g. (**c**) SEM image of the PET@r-GO fiber surface. (**d**) SEM image of the PET@CNTs fiber surface. Data are presented as the mean ± standard deviation from six independently fabricated samples under identical conditions (*n* = 6).

The effect of conductive filler type on the sensing behavior was examined by comparing sensors based on graphene and CNT conductive networks, as shown in Figure 5. Although both types of fillers led to similar overall trends in the *S*–*P* curves, noticeable differences in sensitivity level and decay behavior can be observed.

Within the framework of the proposed model, these differences can be attributed mainly to the contact characteristics between conductive particles. Different filler morphologies, sizes, and surface properties lead to different contact conditions, which most directly affect the contact resistance *R_j_* between particles and are reflected in the variation of the electrical parameter *α*. In addition, the change in filler type may also influence the constant resistance term *K*_2_, such as contributions from relatively stable conductive pathways or electrode interfaces.

As a result, while the general shape of the *S*–*P* curves remains similar, the detailed sensitivity values and their variation with pressure differ for different conductive fillers. This observation indicates that the electrical parameters in the model are able to capture the essential differences introduced by different conductive materials.

### 3.5. Effect of Alkali Treatment

**Figure 6:**
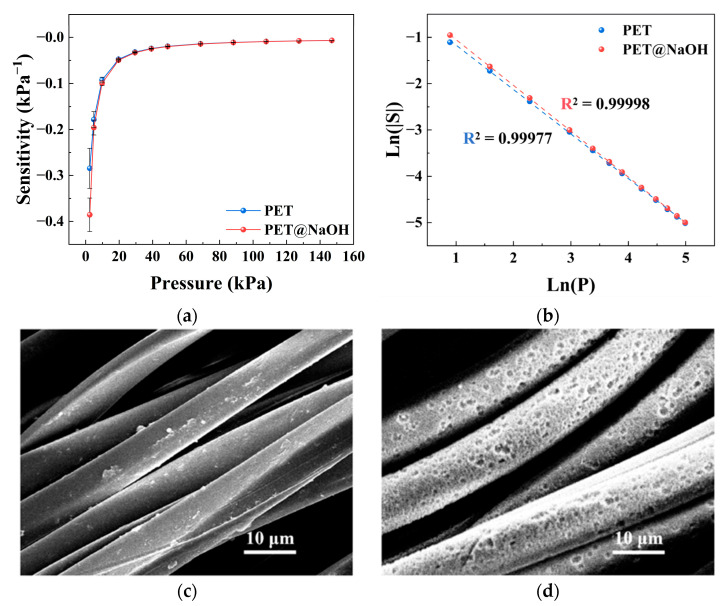
Effect of alkali treatment on the pressure sensitivity of the textile-based sensor. (**a**) Sensitivity–pressure curves of untreated PET and NaOH-treated PET sensors. (**b**) Log–log plots of sensitivity, shown as ln(|*S*|) versus ln(*P*), with linear fitting. (**c**) SEM image of the untreated PET fiber surface. (**d**) SEM image of the NaOH-treated PET fiber surface. Data are presented as the mean ± standard deviation from six independently fabricated samples under identical conditions (*n* = 6).

The effect of alkali treatment on the sensing performance was investigated by comparing untreated and alkali-treated fabrics, as shown in Figure 6. The alkali-treated samples exhibited higher sensitivity in the low-pressure region and a more pronounced decay with increasing pressure.

Within the model framework, this behavior can be understood from both mechanical and electrical aspects. Alkali treatment modifies the fiber surface through etching and roughening, which changes the local deformation behavior of the fiber under radial compression. This effect is mainly reflected in the variation of the effective radial modulus *E_x_*. At the same time, the change in fiber stiffness and surface morphology may also influence the overall compressive response of the textile, and thus affect *E_b_*, leading to a variation in the ratio $E_{b}/E_{x}$. In addition, the roughened surface may improve the interfacial contact between conductive particles and fibers, which can reduce the contact resistance *R_j_* and be reflected in the variation of *α*. As a result, the alkali-treated samples showed higher sensitivity at low pressure, indicating that effective conductive contacts are formed more readily under small loads. This observation is consistent with the model analysis.

### 3.6. Effect of Sensing Area

**Figure 7:**
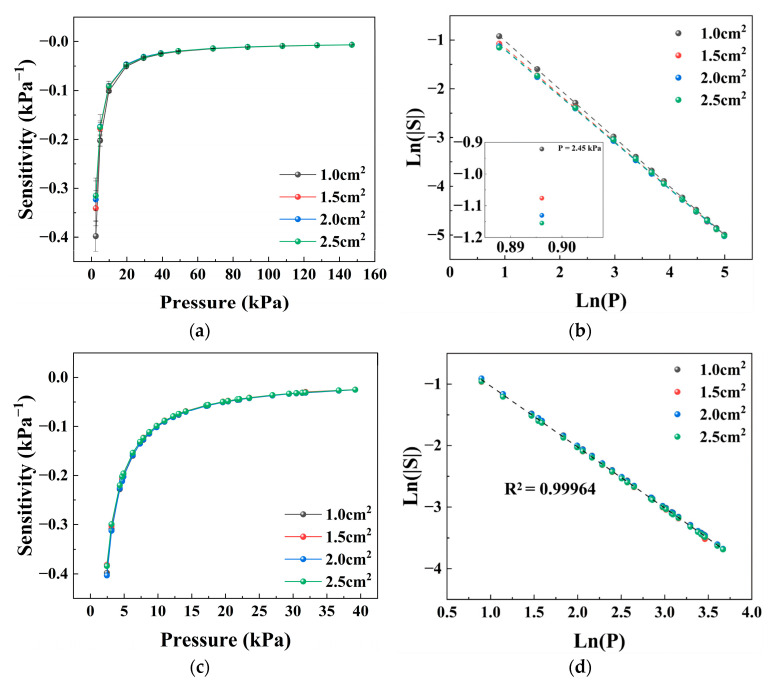
Effect of sensing-layer area on the pressure sensitivity of the textile-based sensor. (**a**) Sensitivity–pressure curves for different sensing areas under uniform loading. (**b**) Logarithmic representation of the corresponding sensitivity curves. (**c**) Sensitivity–pressure curves when the loading area matches the sensing-layer area. (**d**) Logarithmic representation of the corresponding curves. Data are presented as the mean ± standard deviation from six independently fabricated samples under identical conditions (*n* = 6).

The effect of sensing-layer area on the sensor response was examined by varying the size of the conductive textile while keeping the applied loading area constant, as shown in Figure 7. The results indicate that the apparent sensitivity decreases as the sensing-layer area increases under the same loading condition.

It should be noted that the change in sensing-layer area does not primarily correspond to a change in intrinsic material parameters, but rather reflects the matching relationship between the loading area and the sensing area. When the sensing-layer area increases while the loading area remains fixed, the fraction of the conductive network that is effectively involved in the response becomes smaller. As a result, the local resistance change in the compressed region is diluted by the large number of uncompressed conductive paths connected in parallel.

This phenomenon can be understood as a change in the participation ratio of parallel conductive pathways, which leads to a “response dilution effect”. When the loading area matches the sensing-layer area, the differences between samples are significantly reduced, further supporting this interpretation.

### 3.7. Effect of Weave Structure

**Figure 8:**
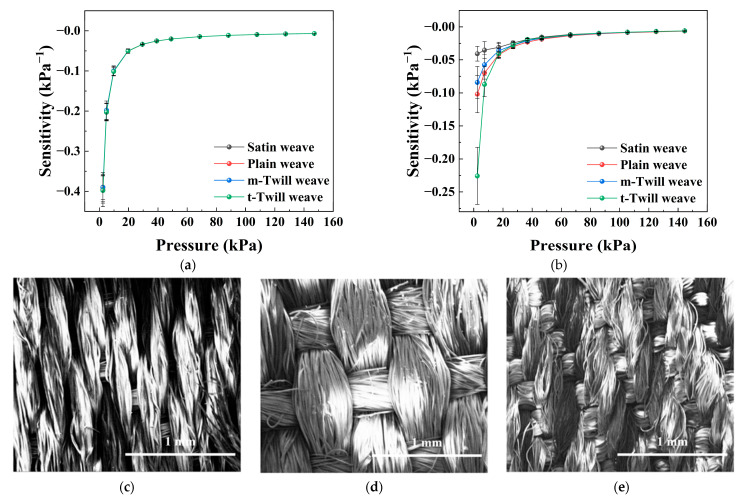
Effect of weave structure on the pressure sensitivity of the textile-based sensor. (**a**) Sensitivity–pressure curves of sensors with different weave structures under an initial preload of 0 g. (**b**) Sensitivity–pressure curves under an initial preload of 25 g. (**c**) SEM image of satin weave. (**d**) SEM image of plain weave. (**e**) SEM image of twill weave. Data are presented as the mean ± standard deviation from six independently fabricated samples under identical conditions (*n* = 6).

The influence of weave structure on the sensing performance was investigated using fabrics with different weave types, as shown in Figure 8. The results show that the differences in sensitivity are relatively small without initial loading, while more distinct differences appear when a preload is applied.

From the model perspective, the weave structure affects both the mechanical response and the topology of the conductive network. Different weave types change the interlacing pattern of fibers and the load transfer path, which significantly influences the effective modulus *E_b_* along the thickness direction. At the same time, the distribution of contact points and conductive pathways is also altered, which can be reflected in the structural factor *K*_1_.

Therefore, the weave structure simultaneously affects the mechanical parameter *E_b_* and the network-related parameter *K*_1_, leading to systematic differences in the shape and slope of the *S*–*P* curves. This indicates that the weave structure is an important structural parameter for tuning both mechanical response and conductive-network characteristics.

### 3.8. Effect of Sensing-Layer Number

**Figure 9:**
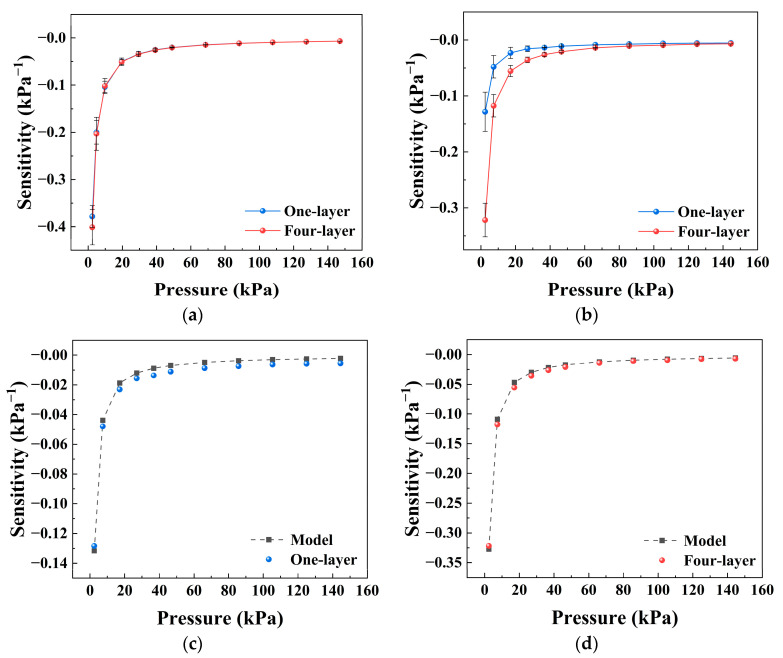
Effect of the number of sensing-layers on the pressure sensitivity of the textile-based sensor and comparison between the model prediction and experimental results. (**a**) Sensitivity–pressure curves of sensors with different sensing-layer numbers under an initial preload of 0 g. (**b**) Sensitivity–pressure curves under an initial preload of 25 g. (**c**) Comparison between experimental data and model prediction for the one-layer structure. (**d**) Comparison between experimental data and model prediction for the four-layer structure. Data are presented as the mean ± standard deviation from six independently fabricated samples under identical conditions (*n* = 6).

The effect of the number of sensing-layers was studied by stacking conductive textiles with different layer numbers, as shown in Figure 9a,b. The results show that the sensitivity behavior changes with the number of layers, and the differences become more pronounced under preload conditions.

Structurally, increasing the number of layers introduces more interfacial contacts in the thickness direction. This effect mainly influences the overall compressive response of the textile, which is reflected in the variation of the effective modulus *E_b_*. Under preload conditions, multilayer structures tend to form more stable interlayer contacts, which modifies the subsequent evolution of conductive pathways during loading. In addition, the change in layer number may also have a secondary influence on the structural factor *K*_1_ and the constant resistance term *K*_2_, for example, by altering the organization of conductive paths and interlayer interfaces. However, the dominant effect of layer number on the *S*–*P* behavior can still be attributed to the variation in *E_b_*.

### 3.9. Comparison Between Model Prediction and Experimental Results

To further evaluate the applicability of the proposed model, the experimental *S*–*P* data were compared with the model predictions, as shown in Figure 9c,d.

Figure 9c presents the comparison for the single-layer structure under a preload of 25 g. The theoretical curve captures the overall trend of the experimental data, showing good agreement within the main pressure range. Figure 9d shows the corresponding result for the four-layer structure, where the model prediction is also consistent with the experimental response.

To quantitatively assess the agreement, the root mean square error (RMSE) and the mean relative error (MRE) were calculated. It should be noted that in the high-pressure region, the sensitivity approaches zero, which leads to a significant amplification of relative error. Therefore, the error analysis was performed within the low- and medium-pressure range.

The RMSE values for the single-layer and four-layer structures were 0.0040 and 0.0056, respectively, while the corresponding MRE values were 27.6% and 13.4%. Although the relative error was higher for the single-layer structure, the model still provided a reasonable description of the overall response trend, and showed better quantitative agreement for the multilayer structure.

The larger relative error in the single-layer case may be attributed to its higher sensitivity to local contact variations. Since the present model adopts a lumped and averaged description, some deviations are inevitable. In contrast, the multilayer structure exhibited more stable contact and load transfer behavior, which is more consistent with the model assumptions.

These results should be interpreted as semi-quantitative trend validation rather than exhaustive statistical benchmarking. The relatively higher MRE in the single-layer case indicates that local contact fluctuations and sample-to-sample variations can still influence the response, which is consistent with the simplified and averaged nature of the model.

### 3.10. Discussion

Based on the above results, although different experimental variables originate from material systems, structural design, or processing conditions, their effects on the *S*–*P* behavior can be interpreted within a unified framework.

Specifically, the conductive particle loading and filler type mainly affect the electrical properties of the conductive network and are reflected in the variation of the parameter *α*. In contrast, factors such as surface treatment, weave structure, and layer number primarily influence the mechanical response of the textile and are associated with changes in $E_{b}/E_{x}$. The sensing-layer area experiment further shows that the macroscopic response is also affected by the participation ratio of conductive pathways.

Therefore, the influence of various structural and material factors can be mapped onto a limited parameter space defined by *α* and $E_{b}/E_{x}$. This mapping provides a unified description of the sensing behavior and helps to understand how different design variables affect the sensor response.

It should also be noted that the present model is based on first-order mechanical simplifications. The Poisson deformation of individual fibers and the anisotropic mechanical response of woven textiles are not explicitly included. In real textile structures, compression in the loading direction may induce the lateral expansion of fibers and further modify the inter-fiber contact area. In the present framework, this indirect lateral interaction is represented in an averaged manner through the effective contact-area evolution and the effective modulus ratio $E_{b}/E_{x}$, rather than being separately described by an explicit Poisson-ratio term. Therefore, the model should be regarded as a simplified explanatory and semi-quantitative framework. Incorporating Poisson deformation and anisotropic textile mechanics will be important for developing a more predictive model in future work.

To further place the present results in the context of recent studies, a comparison between representative flexible textile-based piezoresistive pressure sensors and this work is summarized in Table 1. While the above analysis demonstrates that different structural and material factors can be mapped onto the effective parameters *α* and $E_{b}/E_{x}$, it is also instructive to compare how recent studies describe the sensitivity–pressure behavior.

As shown in Table 1, most recent studies emphasize performance enhancement through material and structural design while relying on empirical fitting or qualitative interpretation to describe the sensing behavior. In contrast, the present work introduces explicit physical parameters α and $E_{b}/E_{x}$ into an analytical framework, allowing different experimental variables to be interpreted within a unified mechanism. This distinction highlights the advantage of the proposed model in providing a physically interpretable reference for sensor design.

## 4. Conclusions

A simplified physical model was established to describe the sensitivity–pressure relationship in textile-based piezoresistive sensors, providing a physically interpretable framework for analyzing their sensing behavior. By idealizing the textile structure as regularly stacked cylindrical fiber units and treating the conductive particles as conductive layers coated on the fiber surface, an analytical expression relating the sensitivity *S* to the applied pressure *P* was derived. The model indicates that the sensitivity is mainly governed by the electrical parameter *α* and the mechanical parameter ratio $E_{b}/E_{x}$, and captures the dominant nonlinear decrease in sensitivity with increasing pressure.

By systematically varying the conductive particle loading, conductive filler type, surface treatment, sensing-layer area, weave structure, and number of sensing-layers, the experimental results show that the response variations caused by different structural and material factors can be reasonably interpreted within the proposed model through changes in *α* or $E_{b}/E_{x}$. This suggests that the model can provide a unified and semi-quantitative description of the main sensing behavior of textile-based piezoresistive pressure sensors. Further comparison between model prediction and experimental data also shows that the model can semi-quantitatively describe the main response trends in the low- and medium-pressure range.

It should be noted that the present model is based on simplified geometric, mechanical, and electrical assumptions. The effective parameters *α*, *E_x_*, and *E_b_* are used as lumped descriptors for mechanistic interpretation and semi-quantitative comparison, rather than as independently measured inputs for strict prediction. In addition, effects such as Poisson deformation, anisotropic textile mechanics, microscopic contact randomness, and coating nonuniformity are not explicitly included in the current framework.

Although the model is based on a simplified representation of the textile structure, the physical relationships revealed here provide useful insight into the structural design and performance optimization of textile-based pressure sensors. More importantly, the introduction of the electrical parameter α and the mechanical parameter ratio $E_{b}/E_{x}$ establishes a unified framework for interpreting the influence of material selection, structural configuration, and processing conditions. This parameter-based description may provide preliminary guidance for the design of customized textile-based pressure sensors in wearable healthcare and soft robotics applications. Future work may incorporate independent mechanical characterization, Poisson deformation, textile anisotropy, microscopic contact randomness, pore structure, and multiscale structural analysis to develop a more predictive quantitative model.

## Figures and Tables

**Table 1:** Comparison of recent flexible textile-based pressure sensors and this work.

Work	Material/System	Main Analysis Method	Explicit Physical Model	Key Physical Parameters	Main Focus	Distinctive Feature
Ref. [7]	Carbonized cotton fabric multilayer flexible pressure sensor	Experimental response characterization	No	–	High sensitivity, wide pressure range, wearable monitoring	Structure-assisted performance enhancement
Ref. [20]	Graphene-impregnated nonwoven textile piezoresistive sensor	Electromechanical characterization and response analysis	No	–	Design/fabrication and sensing behavior	Performance analysis based mainly on experimental behavior
Ref. [21]	MXene–porous polyester textile pressure sensor	Experimental response characterization	No	–	Breathable, high-sensitivity pressure sensing	High device performance and wearable applicability
Ref. [23]	Multilayer textile-material pressure sensor	Device response comparison and performance evaluation	No	–	Multilayer structural design for human–machine interaction	Sensitivity/range optimization through multilayer architecture
This work	rGO/CNT textile-based piezoresistive sensor	Analytical modeling + experimental validation	Yes	$\alpha ,\;E_{b}/E_{x}$	Unified interpretation of *S*–*P* behavior	Physically interpretable framework linking structural/material factors to sensor response

## Data Availability

The datasets presented in this article are not readily available because the data are part of an ongoing study. Requests to access the datasets should be directed to 2242566@mail.dhu.edu.cn.

## Supplementary Materials

The following supporting information can be downloaded at: https://www.mdpi.com/article/10.3390/s26103081/s1. Derivation of the Sensitivity–Pressure Relationship: Figure S1: Idealized parallel fiber stack used for theoretical modeling; Figure S2: Equivalent resistor network representing the conductive pathways in the fiber assembly; Figure S3: Schematic diagram of interfacial axial distance variation with pressure.

## Nomenclature

**Symbol**	**Description**
*S*	Sensitivity
*P*	Applied pressure
*α*	Electrical factor representing contact contribution
*E_x_*	Effective radial modulus of fiber unit
*E_b_*	Effective compressive modulus of textile structure
*A*	Contact area between fibers
*R*	Total resistance
*R_l_*	Contact resistance
*R_j_*	Inter-particle contact resistance
*ρ*	Resistivity of conductive coating
*N*	Effective particle-contact factor
*K*_1_	Geometric factor of conductive network
*K*_2_	Constant resistance term

## References

[1] Barbieri M., Andreoni G. (2024). Textile Pressure Sensors: Innovations and Intellectual Property Landscape. Eng. Proc..

[2] Liu J., Liu H. (2025). Research on Flexible Sensors for Wearable Devices: A Review. Nanomaterials.

[3] Jang H., Lee J., Beak C.-J., Biswas S., Lee S.-H., Kim H. (2025). Flexible Neuromorphic Electronics for Wearable Near-Sensor and In-Sensor Computing Systems. Adv. Mater..

[4] Li J., Zhe L., Yan L., Gang Y., Yan H., Li N., Jian F. (2025). Research progress of machine learning in flexible strain sensors in the context of material intelligence. Mater. Today Phys..

[5] Li S., Yu Y., Yuan S., Li Y., Min T., Kun W., Jie S., Liu Y., Wang H., Li R. (2025). Advances in flexible resistive strain sensors and their applications. Mater. Today Commun..

[6] Zhang Z., Feng B., Yan J., Zhao W., Sun J. (2025). Advances in bio-based wearable flexible sensors. Green Chem..

[7] Yang M., Wang Z., Jia Q., Xiong J., Wang H. (2024). Bio-Skin-Inspired Flexible Pressure Sensor Based on Carbonized Cotton Fabric for Human Activity Monitoring. Sensors.

[8] Schauss G., Hayman A.P.A. (2025). A Taxonomy of Pressure Sensors for Compression Garment Development. Sensors.

[9] Yang K., McErlain-Naylor S., Isaia B., Callaway A., Beeby S. (2024). E-Textiles for Sports and Fitness Sensing: Current State, Challenges, and Future Opportunities. Sensors.

[10] Wu L., Jin Y., Xia Z., Han J., Han W., Dong K., Tang Y., Jiang Q. (2024). Reviews on Flexible Force Sensors Based on Fiber Assemblies with Mass Production Efficiency. Adv. Mater. Technol..

[11] Cui G., Wang C. (2025). Applications and development trends of textile materials in sports: A review. Alex. Eng. J..

[12] Zhao J., Fu K., Wang B., Deng J., Lee H.S., Qiu L., Wang X. (2025). Fabric-based flexible sensors for advanced wearable applications: Development, characterization, and integration into human–machine interaction systems. Flex. Print. Electron..

[13] Li F., Li H. (2025). Recent Advances in Flexible Capacitive Fabric Pressure Sensors: Mechanisms, Materials, and Applications. Chem. Asian J..

[14] Ahmed A., Hasan E.u., Hasseni S.-E.-I. (2025). Smart and Sustainable: A Global Review ofSmart Textiles, IoT Integration, and Human-Centric Design. Sensors.

[15] Liu S., Zhang W., He J., Lu Y., Wu Q., Xing M. (2024). Fabrication Techniques and Sensing Mechanisms of Textile-Based Strain Sensors: From Spatial 1D and 2D Perspectives. Adv. Fiber Mater..

[16] Li X., Wu G.C., Pan C.F., Bao R.R. (2025). Recent Progress in Flexible Sensors Based on2D Materials. J. Semicond..

[17] Qin R., Nong J., Wang K., Liu Y., Zhou S., Hu M., Zhao H., Shan G. (2024). Recent Advances in Flexible Pressure Sensors Based on MXene Materials. Adv. Mater..

[18] Zhang H.-Y., Xiao H., Long J.-J. (2024). Preparation, structure, property and application of MXene in fabricating functional and intelligent textiles: A comprehensive review. J. Mater. Sci. Technol..

[19] Wang P., Hou Z., Chen S., Ren S., Zhao M., Yang L. (2024). Biomaterials for Flexible Pressure Sensors: Innovations and Advancements. J. Mater. Chem. C.

[20] Mahmud M.F., Ahmed M.R., Potluri P., Fernando A. (2025). Understanding the Design and Sensory Behaviour of Graphene-Impregnated Textile-Based Piezoresistive Pressure Sensors. Sensors.

[21] Chen X., Wang C., Wei W., Liu Y., Ge S.S., Zhou L., Kong H. (2025). Flexible and sensitive pressure sensor with enhanced breathability for advanced wearable health monitoring. npj Flex. Electron..

[22] Alhasan S.N., Mirbakht S.S., Guler S., Sahin O., Umar M., Arman Kuzubasoglu B., Yapici M.K. (2025). Artificially Weaved Textile-like Surface Micromachined Graphene-Polymer Flexible Bioelectrodes. Adv. Mater. Technol..

[23] Wang D., Ma G., Zhang X., Zheng K., Zhang J., Ma Z., Han Z., Ren L. (2025). Flexible Pressure Sensor Composed of Multi-Layer Textile Materials for Human-Machine Interaction Applications. ACS Sens..

[24] Chen R., Luo T., Wang J., Wang R., Zhang C., Xie Y., Qin L., Yao H., Zhou W. (2023). Nonlinearity synergy: An elegant strategy for realizing high-sensitivity and wide-linear-range pressure sensing. Nat. Commun..

[25] Ahmad A.U., Qureshi S., Simic M., Mannan H.A., Goyal S., Deepak F.L., Stojanović G.M. (2025). Graphene Nanoplatelet-Nickel Ferrite Coated Textile-Based Embroidered Capacitive Pressure Sensor for Wearable Electronics Application. Sens. Diagn..

[26] Tao Y., Zhang H., Li J., Shi K., Jin L., Guo Y., Shi J. (2025). Earthworm-inspired wrinkled sensors: Ultra-sensitive, flexible, and integrated with deep learning for sound recognition. Chem. Eng. J..

[27] Tan Y., Ivanov K., Mei Z., Li H., Li H., Lubich L., Wang C., Wang L. (2021). A Soft Wearable and Fully-Textile Piezoresistive Sensor for Plantar Pressure Capturing. Micromachines.

[28] Singh L., Tripathy K., Bhattacharjee M. (2023). Nonlinear Response of Polymer-Based Piezoresistive Flexible Tactile Sensor by Modulating Substrate Mechanics. IEEE Sens. J..

[29] Zhou Z., Li Y., Cheng J., Chen S., Hu R., Yan X., Liao X., Xu C., Yu J., Li L. (2018). Supersensitive all-fabric pressure sensors using printed textile electrode arrays for human motion monitoring and human–machine interaction. J. Mater. Chem. C.

[30] Xie J., Pan S., Zhang Y., Wang H., He J., Guo R. (2025). A Flexible Piezoresistive Pressure Sensor Based on Polyester-Spandex Knitted Fabrics for High Performance and Hydrophobicity. Sens. Actuators A Phys..

[31] Laaraibi A.-R.A., Jodin G., Depontailler C., Bideau N., Razan F. (2024). Design and Characterization of Piezoresistive Sensors for Non-Planar Surfaces and Pressure Mapping: A Case Study on Kayak Paddle. Sensors.

[32] Xu H., Gao L., Wang Y., Cao K., Hu X., Wang L., Mu M., Liu M., Zhang H., Wang W. (2020). Flexible Waterproof Piezoresistive Pressure Sensors with Wide Linear Working Range Based on Conductive Fabrics. Nano-Micro Lett..

[33] Liu Q., Dong Y., Mu L., Li J., He J., Liu H., Zhu M., Zhang R., Sun C.-L., Qu M. (2025). High-Performance Textile Piezoresistive Sensor: Graded Contact Hollow Architecture with Synergistic Flame Retardancy, Superhydrophobicity, and Thermal Insulation. ACS Appl. Mater. Interfaces.

[34] Luo Y., Zhao L., Luo G., Dong L., Xia Y., Li M., Li Z., Wang K., Maeda R., Jiang Z. (2023). Highly sensitive piezoresistive and thermally responsive fibrous networks from the in situ growth of PEDOT on MWCNT-decorated electrospun PU fibers for pressure and temperature sensing. Microsyst. Nanoeng..

[35] Li J., Xu B. (2015). Novel highly sensitive and wearable pressure sensors from conductive three-dimensional fabric structures. Smart Mater. Struct..

[36] Zhou Y., He J., Wang H., Qi K., Nan N., You X., Shao W., Wang L., Ding B., Cui S. (2017). Highly sensitive, self-powered and wearable electronic skin based on pressure-sensitive nanofiber woven fabric sensor. Sci. Rep..

[37] Possanzini L., Tessarolo M., Mazzocchetti L., Campari E.G., Fraboni B. (2019). Impact of Fabric Properties on Textile Pressure Sensors Performance. Sensors.

[38] Lian Y., Yu H., Wang M., Yang X., Zhang H. (2020). Ultrasensitive Wearable Pressure Sensors Based on Silver Nanowire-Coated Fabrics. Nanoscale Res. Lett..

[39] Chen Y., Yan X., Zhu Y., Cui M., Kong L., Kuang M., Zhang X., Wang R. (2022). A carbon nanotube-based textile pressure sensor with high-temperature resistance. RSC Adv..

[40] Lee C., Cho C., Park S., Je H. (2026). Highly sensitive, flexible, and biocompatible iontronic pressure sensor based on porous composite electrode for biomechanical signal monitoring. Chem. Eng. J..

