# Supplementary material for: A Simplified Physical Model for the Sensitivity–Pressure Relationship in Textile-Based Piezoresistive Sensors
Source: Sensors (Basel). 2026 May 13;26(10):3081. doi: 10.3390/s26103081 (PMC13210774; doi:10.3390/s26103081)
Supplement: Supplementary file 1 [file sensors-26-03081-s001.zip › sensors-4274872-supplementary.pdf]

Supplementary Materials

Derivation of the Sensitivity–Pressure Relationship

For a flexible textile-based pressure sensor using a woven fabric impregnated with conductive particles as the sensing layer, the relationship between sensitivity S and applied pressure P can be described as follows.

The sensitivity S of a pressure sensor is defined as:

$$S = \frac{\Delta X}{X_0 \cdot \Delta P}$$

where ΔP is the applied pressure variation, X_0 is the initial value before pressure loading, and ΔX is the corresponding change induced by pressure.

In the present system, the electrical response is governed by resistance variation. Therefore, the sensitivity can be expressed as:

$$S = \frac{\Delta R}{R_0 \cdot \Delta P} \tag{S1}$$

Consider a woven textile with a block-like geometry composed of cylindrical fibers impregnated with conductive particles. The textile is assumed to have:

- mass m_0;
- side length a;
- thickness h.

Each fiber is assumed to have:

- diameter d;
- length a;
- density ρ_0.

In this simplified derivation, the textile is represented by an idealized representative volume element with an in-plane side length a and thickness h. The fibers are assumed to run across this representative element, and therefore the effective fiber length within the element is also taken as a.

The conductive particles are assumed to be flake-like, with:

- average mass m_d;
- average thickness d_s;
- average inter-particle contact resistance R_j.

After impregnation, the conductive particles are assumed to form a uniform conductive shell of thickness d_s coating the fiber surface.

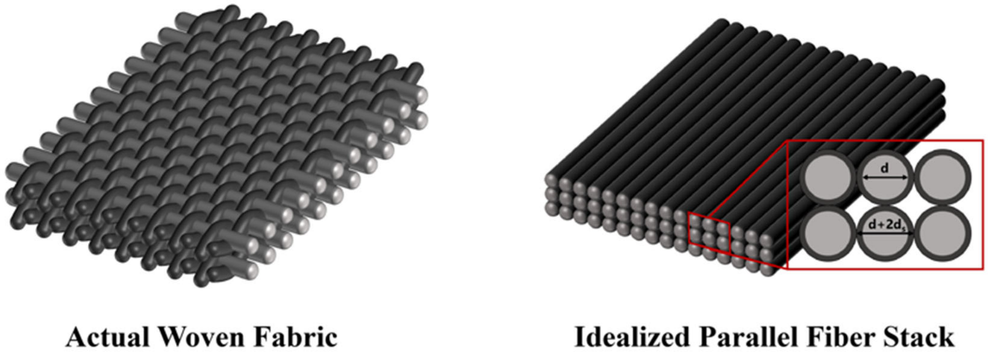

Figure S1. Idealized parallel fiber stack used for theoretical modeling.

For simplicity, the fibers are assumed to be arranged in parallel in a regular packing configuration (as illustrated in Figure S1). The resistance variation originates from:

1. Deformation-induced contact between conductive shells of adjacent fibers;
2. Inter-particle electrical contacts within the conductive network.

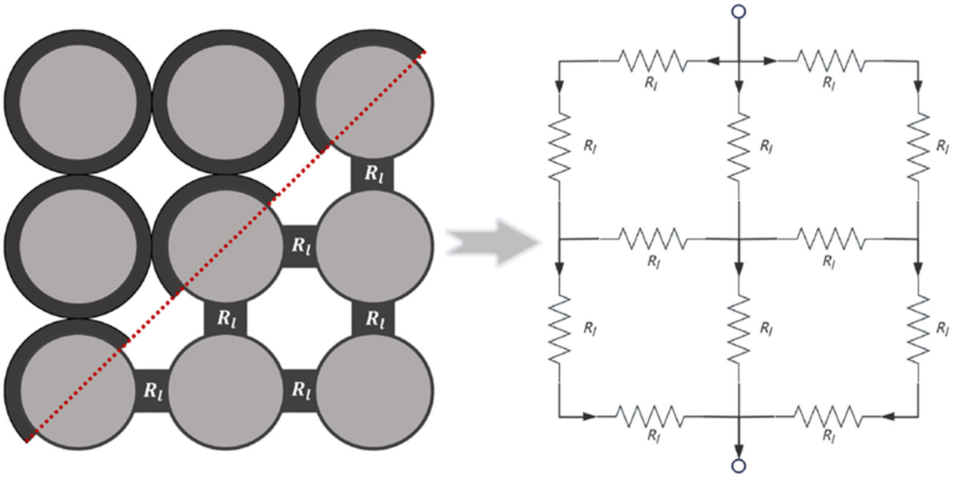

Figure S2. Equivalent resistor network representing the conductive pathways in the fiber assembly.

The conductive particles between two contacting fibers are approximated as an equivalent resistance R_l. These equivalent resistances are connected through conductive paths to form a network, as illustrated in Figure S2.

Each equivalent resistance element can be approximated as a prism with:

- height a;
- width L (approximately between d_s and $2d_s$).

Let the resistivity of this equivalent conductive region be ρ. Then:

$$R_l = \rho \frac{L}{A} + N \cdot R_j \tag{S2}$$

where $A = a \cdot c$, and N is an effective particle-contact factor within the equivalent resistance element.

The total resistance of the textile can be expressed as:

$$R = K_1 \cdot R_l + K_2 \tag{S3}$$

where:

- K_1 depends on the topology of the conductive network;
- K_2 represents other resistive contributions (e.g., electrode resistance), which may be zero.

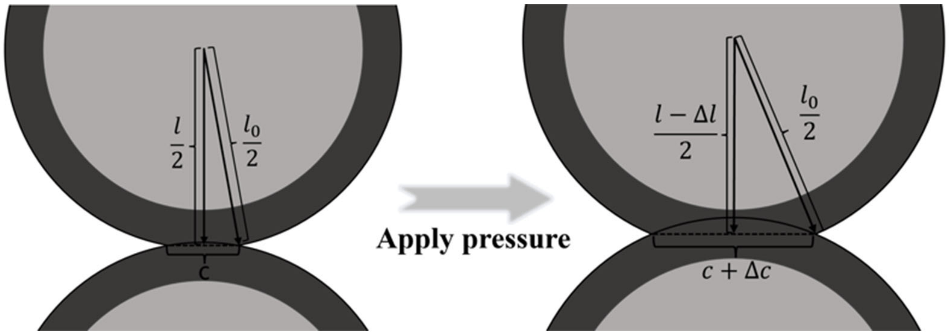

Figure S3. Schematic diagram of interfacial axial distance variation with pressure.

The length c of the equivalent resistive prism changes with deformation induced by the applied force. As illustrated in Figure S3, the longitudinal section of the prism, $A = a{\cdot}c$, is essentially the contact area between two coated fibers.

Let l denote the center-to-center distance between two coated fibers. According to the geometric relation shown in Figure S3, the contact length c can be written as:

$$c = \sqrt{(d+2d_s)^2 - l^2}$$

$$l_0 = d+2d_s$$

Therefore, the contact area A can be expressed as:

$$A = a\sqrt{l_0^2 - l^2} \tag{S4}$$

Differentiating both sides gives:

$$dA = -\frac{a{\cdot}l\,dl}{\sqrt{l_0^2 - l^2}}$$

$$\frac{dA}{A} = -\frac{l\,dl}{l_0^2 - l^2}$$

and thus

$$\frac{dA}{A} = \frac{\frac{2ldl}{l_0^2}}{2\left(\frac{dl}{l_0}\right)^2 + 4{\cdot}\frac{dl}{l_0}} \tag{S5}$$

Next, the relationship between the textile thickness h and the inter-fiber distance l is considered. For the idealized packed structure shown in Figure S1, the total volume of the representative textile element is $h{\cdot}a^2$. This volume is approximated as the sum of the average occupied volumes of n_x coated fibers. Each coated fiber is assigned an effective volume of $a{\cdot}l^2$, where a is the effective fiber length and l^2 represents the averaged transverse area associated with one coated fiber. Therefore,

$$V = a^2{\cdot}h = n_x{\cdot}a{\cdot}l^2$$

$$dh = \frac{n_x}{a}{\cdot}2ldl$$

where n_x is the number of coated fibers in the textile.

Define $E_x = \frac{dP \cdot l}{dl}$ as the effective radial elastic modulus of a coated fiber, and $E_b = \frac{dP \cdot \mathrm{h}}{dh}$ as the effective elastic modulus of the textile in the thickness direction. Substituting the relation between h and l into Equation (S5) yields:

$$\frac{dA}{A} = \frac{\frac{dP}{E_b}}{2\left(\frac{dP}{E_x}\right)^2 + 4 \cdot \frac{dP}{E_x}} \tag{S6}$$

Substituting Equations (S2) and (S3) into Equation (S1), and considering small variations, one has:

$$S = \frac{1}{\left[1 + \frac{A}{\rho L}\left(NR_j + K_2/K_1\right)\right]} \cdot \left(\frac{1}{1 + \frac{dA}{A}} - 1\right) \cdot \frac{1}{dP}$$

Substituting Equation (S6) into the above expression gives:

$$S = -\frac{1}{\left[1 + \frac{A}{\rho L}\left(NR_j + K_2/K_1\right)\right]} \cdot \left(\frac{1}{\left(2\frac{E_b dP}{E_x^2} + 4 \cdot \frac{E_b}{E_x} + 1\right)}\right) \cdot \frac{1}{dP}$$

Let

$$\alpha = \frac{1}{\left[1 + \frac{A}{\rho L}\left(NR_j + K_2/K_1\right)\right]}$$

then

$$S = \frac{-\alpha}{2\frac{E_b}{E_x^2} dP^2 + \left(4 \cdot \frac{E_b}{E_x} + 1\right) dP} \tag{S7}$$

When the initial pressure is taken as zero, one further obtains:

$$S = \frac{-\alpha}{2\frac{E_b}{E_x^2} P^2 + \left(4 \cdot \frac{E_b}{E_x} + 1\right) P}$$

The meanings of N and ρ are discussed below.

The parameter N is introduced as an effective descriptor of the static particle-contact contribution within a single equivalent resistance element. It is related to the number of conductive particles contained in that element, denoted by n_d, but it should not be interpreted as the number of pressure-induced parallel conductive pathways.

Let the total mass of conductive particles loaded into the textile be m_s. Then, the total number of conductive particles is:

$$n_s = \frac{m_s}{m_d}$$

For the packing mode shown above, each conductive fiber is effectively associated with two equivalent resistances. Therefore, the number of conductive particles contained in a single equivalent resistance element can be written as:

$$n_d = \frac{\rho_0 a\pi d^2 \cdot m_s}{8m_0 m_d}$$

Introducing a proportionality constant k, one has:

$$N = kn_d = k\frac{\rho_0 a\pi d^2 \cdot m_s}{8m_0 m_d} \tag{S8}$$

The parameter ρ is defined as the effective resistivity of the conductive coating within a single equivalent resistance element, and it also depends on the conductive-particle loading. In this simplified treatment, higher particle loading is assumed to improve the continuity of the conductive coating and reduce the effective resistivity. This effect is represented through the dependence of ρ on m_s.

Assume that the conductive particle loading required to form a dense conductive shell with thickness d_s on the fiber surface is m_l, and the corresponding resistivity is ρ_l. Then one may write:

$$\rho = \rho_l \cdot \frac{m_l}{m_s} \cdot \frac{L}{A} \tag{S9}$$

Substituting Equations (S8) and (S9) into the expression of α, one obtains:

$$\alpha = \frac{1}{1 + \frac{A}{\rho_l m_l L}\left(\frac{R_j k \rho_0 a\pi d^2}{8m_0 m_d} \cdot m_s^2 + \frac{K_2}{K_1} \cdot m_s\right)}$$

Based on the above derivation, a possible analytical expression relating the sensitivity S of the textile-based pressure sensor to the applied pressure P is obtained. By interpreting this expression, the factors that may affect S and their possible modes of action can be identified, which may provide useful guidance for the fabrication and performance improvement of related textile-based pressure sensors.
